# Neural Component of the Tumor Microenvironment in Pancreatic Ductal Adenocarcinoma

**DOI:** 10.3390/cancers14215246

**Published:** 2022-10-26

**Authors:** Michał Gola, Aleksandra Sejda, Janusz Godlewski, Małgorzata Cieślak, Anna Starzyńska

**Affiliations:** 1 Department of Human Histology and Embryology, Collegium Medicum, School of Medicine, University of Warmia and Mazury, 10-082 Olsztyn, Poland; 2 Department of Pathomorphology and Forensic Medicine, Collegium Medicum, School of Medicine, University of Warmia and Mazury, 18 Żołnierska Street, 10-561 Olsztyn, Poland; 3 Department of Oral Surgery, Medical University of Gdańsk, 7 Dębinki Street, 80-211 Gdańsk, Poland

**Keywords:** pancreatic ductal adenocarcinoma, perineural invasion, tumor innervation, tumor microenvironment

## Abstract

**Simple Summary:**

Pancreatic ductal adenocarcinoma (PDAC) remains one of the most lethal cancers worldwide, with a poor prognosis and an increasing incidence. Recently, the tumor microenvironment, including its neural component, has gained the attention of cancer researchers. Neural regulation of pancreatic cancer carcinogenesis is, however, poorly understood. Only lately have the diversified autonomic nerve fibers been noticed in cancer research. Targeting sympathetic and parasympathetic nerves in various malignancies, including PDAC, may bring new therapies into clinical practice. Omnipresent perineural invasion in pancreatic cancer is associated with a poor prognosis. Moreover, novel quantification with a distinction between perineural and endoneural invasion could help stratify the risk of relapse and mortality for patients with this cancer. Newly described biologic phenomena—cancer-related axonogenesis and neurogenesis—are understudied in pancreatic cancer. This review aims to summarize and integrate the role of nerves in PDAC.

**Abstract:**

Pancreatic ductal adenocarcinoma (PDAC) is a highly aggressive primary malignancy of the pancreas, with a dismal prognosis and limited treatment options. It possesses a unique tumor microenvironment (TME), generating dense stroma with complex elements cross-talking with each other to promote tumor growth and progression. Diversified neural components makes for not having a full understanding of their influence on its aggressive behavior. The aim of the study was to summarize and integrate the role of nerves in the pancreatic tumor microenvironment. The role of autonomic nerve fibers on PDAC development has been recently studied, which resulted in considering the targeting of sympathetic and parasympathetic pathways as a novel treatment opportunity. Perineural invasion (PNI) is commonly found in PDAC. As the severity of the PNI correlates with a poorer prognosis, new quantification of this phenomenon, distinguishing between perineural and endoneural invasion, could feature in routine pathological examination. The concepts of cancer-related neurogenesis and axonogenesis in PDAC are understudied; so, further research in this field may be warranted. A better understanding of the interdependence between the neural component and cancer cells in the PDAC microenvironment could bring new nerve-oriented treatment options into clinical practice and improve outcomes in patients with pancreatic cancer. In this review, we aim to summarize and integrate the current state of knowledge and future challenges concerning nerve–cancer interactions in PDAC.

## 1. Introduction

Currently, an increasing number of studies concerning different malignancies are focused on the role of the tumor microenvironment (TME) in cancerogenesis. TME is a complex idea of the internal cancer environment, referring to the coexistence of various types of cells with the surrounding tissue [1]. Traditionally, it is divided into three constituents: stroma, cellular elements, and soluble proteins such as cytokines, chemokines, and growth-regulating factors [2]. The extracellular matrix (ECM) is an acellular scaffolding that is a complex network of structural, specialized macromolecules, such as collagen, elastin, fibronectin, laminin, and proteoglycans. Furthermore, cancer-associated fibroblasts, Schwann cells, adipocytes, mesenchymal stem cells, immune cells, extravasated blood cells, and neurons are acknowledged inhabitants of the tumor milieu [3,4]. In addition, pancreatic stellate cells (PSCs) are likely to be the most crucial residents of the PDAC microenvironment [5,6]. Multiple studies have shown that interaction between cancerous and nonmalignant cells, both creating a tumor microenvironment, determine a tumor’s proliferation and progression [4]. Furthermore, complex crosstalk among the cellular and molecular components of the TME may cause chemotherapy resistance [7,8].

Pancreatic ductal adenocarcinoma possesses an incredibly complex tumor microenvironment, which is responsible for its highly aggressive nature. Dense desmoplastic stroma is a characteristic feature of PDAC, predominantly composed of various groups of cells, including stellate cells, endothelial cells, nerves, immune cells, and ECM [7,9,10]. Desmoplasia results in forming a mechanical barrier around PDAC cells, thus reducing chemotherapeutic agents’ availability in the TME, and, additionally, causing a lack of immune cells in the tumor milieu, which reflects both chemoresistance and poor anticancer immune response [8,10,11].

PDAC, arising in the exocrine pancreas, is one of the most aggressive malignancies, with a poor overall prognosis: the overall 5-year survival rate is <10% [12,13]. PDAC is estimated to be the second leading cause of cancer-related death worldwide in 2030 [14]. The majority of people are diagnosed with an unresectable stage [15], even though 20% of patients with a PDAC undergo surgical resection. However, cancer recurrence is common in this group; thus, a plethora of people with a PDAC diagnosis will ultimately lose the fight against the disease [16,17]. It is essential, therefore, to search for new treatment modalities. A deeper focus on the pancreatic TME, including its neural component, may bring novel predictive factors and targeted therapies.

The impact of the nervous system as an integral part of the TME on cancerogenesis has recently gained well-deserved attention. An increasing amount of evidence suggests a pivotal role of the autonomic nervous system in tumor growth and progression [18,19,20].

Neurons in general are required during embryonic development, for tissue repair and regeneration. First mentioned nearly 200 years ago, nerve dependency was observed among salamanders in which denervation of the amputated limb inhibited its regeneration [21]. In the past, nerves were believed to be inert bystanders in cancers. In the last decade, ground-breaking studies have shown that not only is the neural component far from being passive, but ample evidence indicates that nerves are major contributors to cancer development and its progression.

Chronic stimulation of the sympathetic nervous system was found to drive tumorigenesis and cancer progression via increased levels of catecholamines [22]. Surprisingly, parasympathetic nerves possess a presumably inhibitory role in PDAC and behave in an opposite manner to that observed in gastric and prostate cancers [23,24,25,26]. In PDAC, cholinergic nerves can inhibit the growth of cancer stem cells (CSCs) and have a suppressing influence on liver metastases [26]. Using drugs that block sympathetic and parasympathetic transmission could serve in favor of inhibiting tumor progression [27,28].

Evidence proving cancer’s nerve-dependency is abundant. Novel data suggest that both neoneurogenesis—activation of nerve growth in tumor stroma—and axonogenesis—the axonal outgrowth from pre-existing nerves—can play a crucial role in cancer progression [17,29,30,31]. However, nerve–cancer cell relations are still understudied and more research to decipher the role of neuro- and axonogenesis needs to be conducted.

The other topic is the role of perineural invasion (PNI) in tumor progression. In this process, neoplastic cells can migrate along nerves. Neoplastic invasion of the nerves corresponds to increased cancer growth and poorer patient outcome in a variety of malignancies, including prostate cancer, head and neck cancer, and gastric cancer [32]. PNI is observed in 75% of resected cancers and it seems to be the most important way of extracapsular spread in this malignancy [33,34].

In this review, we attempt to explain the role of the neural component of the tumor microenvironment in pancreatic ductal adenocarcinoma. The present work may help elucidate how nerve dependency can affect both research and clinical practice.

## 2. Pancreas—Basic Relationships

The pancreas is a mixed exocrine–endocrine, lobulated gland that produces digestive enzymes and hormones. The exocrine part, which accounts for more than 95% of the pancreatic mass, is composed of densely packed acini, which comprise acinar cells drained by intercalated ducts formed by centroacinar and ductal cells, with the associated connective tissue, vessels, and nerves. Acinar cells produce digestive enzymes that are released into intercalated ducts, where they are mixed with bicarbonate-rich fluid secreted by centroacinar and ductal cells. Assorted fluids enter the intralobular, interlobular, and main pancreatic ducts to eventually reach the duodenum via the major or minor papillae. Approximately 1–2% of the pancreatic mass is formed by the endocrine part composed of Langerhans islets. There are mostly four prevalent cell types forming islets: β cells (secreting insulin), α cells (secreting glucagon), δ cells (secreting somatostatin), and PP cells, which secrete pancreatic polypeptide. Additionally, isolated islet cells can be found dispersed in the acinar lobules or in association with ducts.

There is manifold evidence of complex exocrine–endocrine crosstalk occurring in an endocrine, paracrine, and autocrine manner [35]. The main neurotransmitters controlling the islet–acinar axis are acetylcholine (ACh) and norepinephrine (NE). They act cooperatively with various co-transmitters and neuropeptides, such as nitric oxide, vasoactive intestinal polypeptide, calcitonin gene-related peptide, neuropeptide Y, substance P, and galanin [36]. However, a plethora of agents controlling the intra-pancreatic islet–acinar axis is secreted by the islets themselves. Insulin, glucagon, somatostatin, and ghrelin are examples of islet-derived hormones that modulate the exocrine and endocrine interplay. It has been shown that insulin exerts a trophic effect on the exocrine pancreas [37,38], whereas other islet-derived hormones have demonstrated inhibitory effects on exocrine secretion [39,40].

Within the pancreas, the third fundamental component is the stroma, possessing multiple interesting elements.

Among the various stromal cells, pancreatic stellate cells (PSCs) can be found in the exocrine regions of the pancreas [41]. PSCs occur in two main phenotypes: quiescent and activated. Quiescent PSCs play a role in maintaining a normal tissue architecture by regulating ECM turnover [42], whereas activated PSCs’ role is multidimensional. In PDAC they contribute to desmoplastic stroma formation, as well as tumorigenesis and tumor progression [8,10,11,43]. Furthermore, PSC-derived IL-6 induces epithelial–mesenchymal transition (EMT) in a paracrine fashion via the STAT3/NRF2 pathway [44]. Interestingly, PSCs were found to co-travel with pancreatic cancer cells (PCCs) to form distant metastases [45,46]. In addition, it has been recently suggested that PSCs play an essential role in PDAC-related pain [47]. Novel findings show that PSCs via Sonic hedgehog and hepatocyte growth factor/c-Met signaling pathways promote PNI in PDAC by activating the mTOR/NGF axis [48,49,50].

Telocytes, a relatively newly described group of stromal cells, may be found in the exocrine pancreas [51], in close proximity to the acinar and ductal cells, where they form a broad network of homo- and heterocellular connections [51]. Telocytes possess spindle-shaped cell bodies and a few exceptionally long cell prolongations known as telopodes [51]. Interestingly, as telocytes release microvesicles, they are thought to be involved in contactless intercellular cross-talk [51]. In hepatocellular carcinoma, telocytes were found to promote metastasis by the production and secretion of matrix metalloproteinase 9 [52].

Moreover, enterochromaffin cells sparsely surround the ductal system of the pancreas, exerting inhibitory effects on pancreatic fluid secretion [53].

Pancreatic ECM contains numerous exosomes, which are membrane-bound extracellular vesicles released into the TME by the majority, if not all, cells, transporting proteins, mRNAs, miRNAs, fragments of DNA, and lipids [54,55]. As exosomes cargo neurotrophic factors, they are thought to promote axonogenesis [30,56]. Novel data have also shown that exosomes may drive sensory-to-adrenergic nerve transdifferentiation [31].

## 3. Physiologic Innervation of the Pancreas

The pancreas is abundantly innervated by afferent and efferent nerve fibers, engaging both the autonomic and central nervous system. The extrinsic autonomic nerve fibers stem from the vagus nerve and providing sympathetic innervation splanchnic nerves, also carrying sensory nerve fibers from the dorsal root ganglion (DRG) and ganglia of the sympathetic chain. The head of the pancreas is believed to be the most innervated part of this organ [57,58]. Both parasympathetic and sympathetic innervations are likely to decrease from the head to the tail of the pancreas [59,60,61].

The vagus nerve fibers can both directly enter the pancreas or by going through the coeliac trunk without forming a synapse, to eventually reach the intrapancreatic intrinsic ganglia, mainly surrounding the Langerhans islets. Nevertheless, parasympathetic innervation of human islets is sparse [62]. The vagus nerve is known to affect both exocrine and endocrine secretion. Acetylcholine acting on the M1 and M3 muscarinic receptors on the acinar cells influences pancreatic enzyme secretion [63,64]. Additionally, parasympathetic stimulation evokes fluid and HCO_3_^−^ secretion. The main source of ACh is the Langerhans islets’ α cells rather than neuron fibers [62,65]. The α-cell-derived ACh directly stimulates insulin secretion, simultaneously providing inhibition of insulin secretion indirectly via δ-cell-derived somatostatin [62,65]. In addition, multiple non-adrenergic, non-cholinergic neurotransmitters, such as nitric oxide, vasoactive intestinal polypeptide, or pituitary adenylate cyclase-activating polypeptide, contribute to excitatory vagal-like protein secretion [66,67]. Pancreatic enzyme secretion and release of gut hormones in the cephalic phase of gastric secretion depend entirely on the vagus nerve [68,69].

Adrenergic neurons innervate the intrapancreatic ganglia, as well as the islets, blood vessels, and, to a lower degree, the exocrine part of the pancreas. Noradrenaline acting on α- and β-adrenoreceptors, along with neuropeptide Y and galanin are all executors released by the postganglionic sympathetic neurons [70,71,72]. Interestingly, single-cell RNA sequencing analyses provided insights into the cellular and molecular diversity of sympathetic neurons. Five subtypes of noradrenergic neurons have thereby been identified in mouse stellate and thoracic ganglia [73].

Sympathetic nervous control of both exocrine and endocrine pancreas is, however, rather indirect, as the adrenergic fibers contact endocrine, as well as acinar and ductal cells, to a small extent [74]. Instead, their axons reach the abundant contractile smooth muscle cells of the blood vessels. In this manner, sympathetic input influences local blood flow in response to catecholamines. NE-induced vasoconstriction has been shown to reduce perfusion of the human islets, thereby reducing the insulin release into circulation [75]. Similarly, reduced blood flow diminishes fluid secretion by the exocrine pancreas [66].

**Figure 1:**
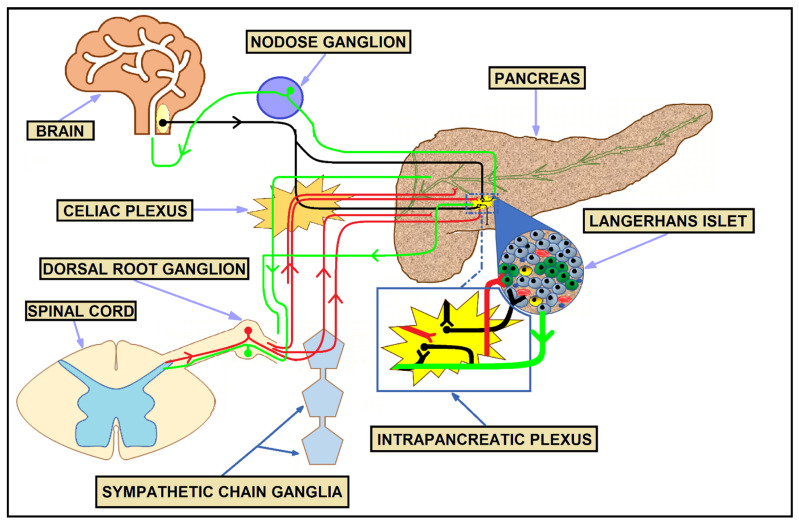
Autonomic and sensory innervation of the healthy pancreas. Green nerve cells—sympathetic neurons; red nerve cells—sympathetic neurons; black nerve cells—parasympathetic neurons.

Pancreatic sensory information is conveyed to the central nervous system via both parasympathetic (vagal) or sympathetic (spinal) pathways. Both vagal and spinal afferent fibers are composed of either unmyelinated C or small-diameter myelinated Aδ fibers and their cell bodies are located in the DRG and nodose ganglia, respectively. Abundant afferent input is involved in sensing both mechanical and chemical signals via a wide range of specific receptors and ion channels, including the transient receptor potential vanilloid 1 (TRPV1), which mediates the release of proinflammatory neuropeptides such as calcitonin gene-related peptide and substance-P, facilitating pain transmission [76]. Figure 1 describes different components of the healthy pancreas innervation.

## 4. Innervation in PDAC

### 4.1. Parasympathetic Innervation

The precise role of the parasympathetic nerve fibers in PDAC is still undetermined. Experimental studies in mice connect the high vagal nerve activity with reduced PDAC progression [77]. Novel data obtained from the Kras oncogenic mouse model have shown that subdiaphragmatic vagotomy advances PDAC development [26]. Mice that underwent vagotomy at 8 weeks of age developed a larger area of pancreatic intraepithelial neoplasia (PanIN) measured at 20 weeks compared to mice with an intact vagus nerve [26]. Furthermore, vagotomy of mice correlated with increased expression of the M1 receptor in the murine pancreatic tissue [26]. Moreover, the addition of a nonselective muscarinic agonist such as bethanechol to the gemcitabine monotherapy of vagotomized mice with established PDAC extended their overall survival from 29 to 48 days (*p* < 0.001), most likely via suppression of the CSC compartment [26]. Further findings revealed that M1 receptor stimulation downregulates EGFR/MAPK and PI3K/AKT signaling, thus suppressing tumorigenesis in PDAC [26]. The cholinergic transmission was also found to diminish metastatic growth in the liver [26].

Altogether, it may suggest that vagal signaling has a significant inhibitory effect on PDAC and that cholinergic agonists could be helpful in the treatment of PDAC at both early and late stages. It is crucial to mention that bethanechol stimulates, in fact, only muscarinic receptors, whereas, ACh released from the vagal nerve endings, acts on both muscarinic and nicotinic receptors as an agonist. Moreover, it has been demonstrated that ACh helps create immunosuppressive TME in PDAC [78]. Surprisingly, ACh, by stimulating nicotinic receptors, increases the levels of local catecholamines [79].

Both normal pancreatic duct epithelial cells and PDAC cells express an autocrine catecholamine loop that appears to stimulate their proliferation acting on nicotinic ACh α3, α5, α7, and beta-adrenergic receptors [79]. Acting on nicotinic ACh receptors, especially its alpha7 subunit, nicotine promotes EMT via the hypoxia-inducible 1α factor/yes-associated protein 1 positive feedback loop [80]. Enhanced levels of yes-associated protein 1 and hypoxia-inducible 1α factor have been shown to induce EMT and drive tumorigenesis in PDAC cells in vitro and in murine xenograft models [80]. Interestingly, the M4 muscarinic receptor has been found to be the predominant receptor among all muscarinic receptor types expressed in human PDAC cells [26].

In fact, the vagus nerve as a whole is likely to inhibit sympathetic neural activity. Consequently, a subdiaphragmatic vagotomy can liquidate its inhibitory impact on adrenergic signaling, causing elevated plasma adrenalin and noradrenalin levels shortly after surgery and in a chronic fashion [81,82]. These findings emphasize the complexity of parasympathetic innervation in the PDAC microenvironment. It is likely that the cholinergic output is both stimulatory and inhibitory in PDAC tumorigenesis at the same time, with a predominance of the antitumorigenic component.

In order to obtain data about the parasympathetic influence in PDAC, more sophisticated research needs to be conducted. For instance, an effective distinction between nicotinic and muscarinic effects on PDAC may be achieved by selective vagotomy, which could possibly be done by surgical or pharmacological methods. Using selective muscarinic or nicotinic receptor agonists/antagonists in experimental research could help decipher the exact role of each type of receptor localized at particular cells in pancreatic TME. Ultimately, translation into broader use of old and well-known drugs targeting the parasympathetic nervous system as an adjuvant in PDAC treatment could be attained.

### 4.2. Sympathetic Innervation

In general, sympathetic output is believed to stimulate PDAC development [83,84]. However, some recent studies suggest the opposite [85,86]. In the PDAC microenvironment, both cancer and diverse stromal cells possess β-adrenergic receptors on their cell surface [83,87]. In the PDAC cells membrane, both β1- and β2-adrenoreceptors were detected, with β2 receptors predominating over β1 [83].

Noradrenaline, which is believed to be a “stress hormone”, plays a crucial role in chronic stress; consequently, it may promote the development and other malignant biological behaviors of PDAC acting on the β2-adrenoreceptors upregulated in pancreatic cancer tissue [84,88]. Notably, NE itself has been found to be overexpressed in pancreatic tumor tissue while being barely detectable in tumor-adjacent tissues [89]. Noteworthy, PDAC cells synthesize their own NE and adrenaline, thus forming the autocrine catecholamine loop that stimulates their proliferation and local NE accumulation [79,90]. Furthermore, NE enhances cell viability and inhibition of apoptosis via several pathways acting harmoniously, such as P38/MAPK and Notch-1 pathways, cAMP response element-binding protein, and nuclear factor-κB [91,92,93]. Additionally, NE-mediated activation of STAT3 was found to upregulate nerve growth factor (NGF) and matrix metalloproteinase 2 and 9 expressions, thus promoting PDAC cells’ invasiveness, migratory ability, and PNI formation [94].

Novel in vitro results suggest that catecholamines through the β2-adrenoreceptor and PKA/ERK pathway induce enhanced secretion of neurotrophins, such as NGF and brain-derived neurotrophic factor, from human PDAC cells. As a result, increased nerve–cancer crosstalk causes neurite outgrowth toward the cancer colony, occurrence of prominent PNI, and enlarged intratumoral nerves in a pancreatic TME [90].

A recent study by Guillot et al. revealed a cancer-protective function of sympathetic nerves in PDAC [86]. In this study, sympathectomized mice exhibited an increased intratumoral CD163+ macrophage population, which were found to be protumorigenic and immunosuppressive [86].

Such a broad catecholamine-cancer growth dependence has led to questions such as if the β-blocker treatment of patients prior to diagnosis or with active PDAC disease may contribute to the decreased pancreatic cancer risk or improved survival. A few preclinical and clinical data have targeted these questions, but with different results. Preclinical studies suggest that treatment with selective β2-adrenoreceptor antagonists may be more beneficial than with more commonly used selective β1-blockers [93,95]. Recently, one nested case–control study assessed the outcomes of β-blockers use on PDAC risk [96], and, consistent with the results a large prospective cohort study [97], no significant risk reduction for developing pancreatic cancer among patients using any β-blocker was found. However, analysis by receptor selectivity demonstrated a diminished risk of PDAC development among patients treated with non-selective β-blockers for more than two years [96].

Results regarding the overall survival of patients with PDAC who used β-blockers were inconsistent [98,99]. A Swedish general population-based cohort study demonstrated that β-blocker use may have a beneficial effect on survival among PDAC patients [98]. Nevertheless, a US-based epidemiological study determined that no β-blocker treatment, even after stratification by receptor selectivity, lasting for six months before diagnosis, improved survival in pancreatic cancer [99]. However, according to the same study, continuous β-blocker usage within 12 months surrounding PDAC diagnosis conferred significant improvement in overall survival [99].

Taken together, further experimental studies are needed to confirm the possible advantages and disadvantages of sympathetic and neurotrophin signaling inhibition. As data revealed both cancer-protective and protumorigenic functions of the sympathetic input, the categorization of neurons into molecularly distinct subtypes in relation to their functional diversity could be meaningful [73]. Furthermore, more high-quality studies, including randomized controlled trials, need to be conducted to establish beta-blockers as potential chemo-preventive and/or novel alternative for cancer adjuvant chemotherapy in PDAC.

### 4.3. Sensory Innervation

In PDAC, sensory nerves are believed to contribute to pancreatic cancer initiation and progression [100]. It has been well documented that substance P, released from sensory nerve endings, contributes to pain sensation; however, novel findings highlight its role in PDAC progression as well [47,101,102]. Data acquired from the murine PDAC model demonstrate that precancerous PanIN lesions are accompanied by increased sensory innervation [103]. In vitro findings suggest that PanIN cells may actively recruit sensory axons in their close proximity [104]. Sensory nerves ablation in a genetically engineered mouse PDAC model slowed the development of PanIN lesions and significantly prolonged overall survival [100]. Interestingly, in both murine and human PDAC models, sensory nerves promoted the proliferation of neuroendocrine PanIN cells via substance-P/neurokinin 1 receptor signaling and JAK–STAT pathway activation [104]. It has also been suggested that substance-P/neurokinin 1 receptor signaling plays a crucial role in the development of metastasis and the PNI occurrence in PDAC [101]. Thus, targeting sensory nerves or blocking the substance-P/neurokinin 1 receptor signaling may be efficacious in terms of increased overall survival time and better pain management among people at various stages of PDAC.

**Figure 2:**
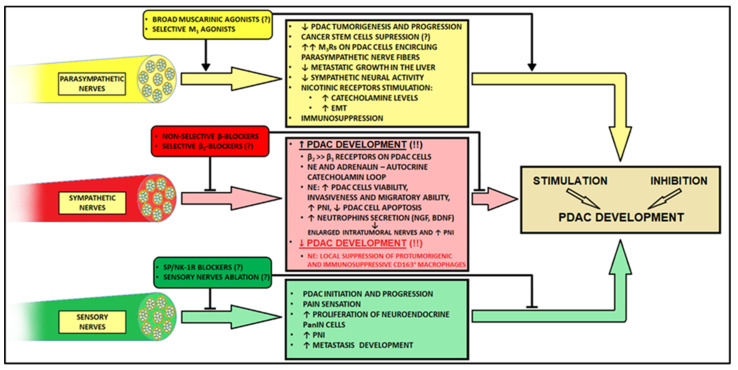
The complexity and impact of the autonomic nervous system on the PDAC tumor microenvironment and possible nerve-oriented treatment modalities. SP/NK-1R, substance-P/neurokinin 1 receptor; PDAC, pancreatic ductal adenocarcinoma; NE, noradrenaline; NGF, nerve growth factor; BDNF, brain-derived neurotrophic factor; PNI, perineural invasion; PanIN, pancreatic intraepithelial neoplasia.

Figure 2 summarizes the role of particular nerve fibers in PDAC tumorigenesis.

## 5. Perineural and Endoneural Invasion

Perineural invasion is present in various solid tumors, such as prostate, head and neck, and biliary tract cancers [105,106,107,108]. It is considered a frequent event in PDAC, as its prevalence varies between 70% and 100% among studies [32,109,110].

**Figure 3:**
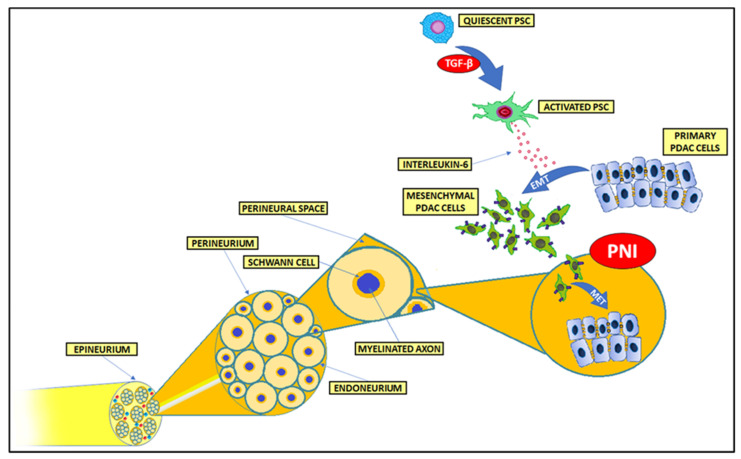
Schematic presentation of the structure of a peripheral nerve. Primary PDAC cells undergo EMT and in a mesenchymal state invade the perineural space of autonomic nerves, where MET-like changes in PDAC cells promote tumor colonization within the nerve. PDAC, pancreatic ductal adenocarcinoma; EMT, epithelial–mesenchymal transition; MET, mesenchymal–epithelial transition; PNI, perineural invasion; PSC, pancreatic stellate cell.

Although PNI was firstly described in 1985 by Batsakis [111], the neoplastic invasion of nerves in PDAC was already observed in 1944 [112]. Batsakis described PNI as neoplastic cell invasion in, around, and through nerves. Throughout the years, however, plenty of PNI definitions have been proposed [113,114]. Liebig et al. in 2009 proposed the most commonly used one, according to which PNI may be described as the existence of cancer cells within any of the three nerve sheath layers or invasion of neoplastic cells in close proximity to the nerve with involvement of at least one-third of its circumference [115]. The structural organization of peripheral nerves is shown in Figure 3. It has been proposed to distinguish the tumor cells’ infiltration of the perineurial space from the extension of the neoplastic cells along the outer surfaces of nerves [106,116,117]. Additionally, further divisions of the PNI phenomenon have been described, distinguishing between PNI and endoneural (intraneural) invasion (ENI) [117,118]. ENI is perceived as the infiltration of cancer cells into the endoneurium, where they are present within the nerve fascicles, affecting Schwann cells and the general nerve microenvironment [118,119,120]. In particular, Ceyhan et al. found that amidst 149 PDAC cases, 115 had PNI (77%), among which only 63 possessed more severe ENI (42%) [118]. It has been shown that patients possessing ENI had more severe and frequent pain sensation than patients with only PNI [118].

In some cancers, such as prostatic cancer or adenoid cystic carcinoma of salivary glands, the PNI phenomenon can constitute the dominant way of metastasis [119,120]. Ubiquitous PNI occurrence has been established as an independent factor of poor prognosis in several malignancies, such as colorectal, gastric, prostatic, biliary tract, and head and neck tumors [106,108,121,122,123,124,125,126]. Data from PDAC patients also link PNI presence to unfavorable outcomes. [109]. It has been shown that cancer recurrence depends on the depth of the tumor cells’ nerve invasion, as ENI was more noxious than PNI and caused diminished median disease-free survival and overall survival among positive PDAC cases (disease-free survival: 13.4 and 32.9 months; overall survival: 28.1 and 45.7 months, respectively) [127]. Local/distant recurrence was remarkably higher comparing ENI (94.3%) to PNI (71.6%) [127]. Noteworthy, a retrospective study of 153 PDAC cases determined that the degree of intrapancreatic nerve invasion may be useful as a predictor for the recurrence of disease after surgery [128].

Intriguingly, one retrospective study found a difference in the frequency of PNI between patients who received neoadjuvant therapy and patients who did not receive any form of neoadjuvant therapy [127]. In a cohort of 212 patients with PDAC who received neoadjuvant therapy, PNI occurred in 123 (58%) cases and ENI in 35 (28.5%) patients [127]. However, among the 60 patients who did not have neoadjuvant therapy, PNI was present in 80% [127]. Barbier et al. have shown a similar trend concerning PNI among patients with PDAC who did or did not receive neoadjuvant therapy prior to surgery (43% to 93%, respectively) [129]. It is important to note that, in PDAC, PNI presence in the treated group correlated with a larger tumor size, resection margin status, lymph node metastasis, and a post-neoadjuvant pathologic tumor stage [127]. Moreover, novel meta-analysis data showed that PNI was also significantly associated with an increased risk of peritoneal dissemination [130].

Interestingly, PNI is likely to change the proportion of sympathetic and parasympathetic nerve fibers within the invaded nerves [131]. Tumor-occupied nerves were shown to have decreased amounts of both noradrenergic and cholinergic fibers; however, it has not been established whether PDAC cells tend to invade nerves with a low content of sympathetic and parasympathetic components at baseline or whether they induce downregulation of sympathetic and cholinergic fibers in situ [131].

At the molecular level, multiple cytokines, chemokines, and adhesion molecules are believed to promote PNI [132]. It was recently shown that sensory nerve-derived chemokines, such as CCL21 and CXCL10, and their receptors CCR7 and CXCR3, are important at the early stages of PNI formation [133]. They are believed to play a role in the attraction and migration of PDAC cells towards peripheral nerves, thus promoting neural remodeling and cancer pain. The presence of ENI positively correlated with the high expression of CCR7 [133]. Several studies have also linked axon guidance molecules such as semaphorin 3D, plexin D1, Slit glycoprotein, and the Roundabout signaling pathway with PNI occurrence [134,135]. Noteworthy, the NF-kappa B pathway, crucial for the initiation and progression of PDAC, has been found to play an essential role in PNI, as well as in EMT induction [136,137]. Thus, novel NF-kappa B pathway inhibitors (triptolide and its prodrug, Minnelide™) have been used with promising effects in vitro and in vivo murine models to diminish tumor–nerve crosstalk, PNI, EMT, and metastasis formation [138]. Moreover, novel data revealed the interleukin-6/GP130 axis as a stimulator of PNI in PDAC [139].

It is worth mentioning that in colorectal cancer exosomal transfer is thought to be closely related to PNI presence [140]. Ultimately, deciphering the exact role of exosomes in PNI formation in PDAC may be possible.

## 6. Tumor–Nerves Bidirectional Interactions—Axonogenesis, Neurogenesis, and Nerve Reprogramming

Cancer–nerve dependence manifests itself distinctively in neurogenesis, which refers to the formation of new functional neurons from neural precursors, and axonogenesis defined as a cancer-induced axonal outgrowth from pre-existing nerves, resulting in an increased nerve density and nerve phenotype reprogramming. Constantly, there is still a dispute about where the new neural cells may originate from.

One opinion suggests that human CSCs derived from both gastric and colorectal cancer are able to differentiate into functional neurons in vitro and in vivo [141]. Among the differentiated CSCs, both parasympathetic and sympathetic neurons are found. It has been proposed that de novo formed neurons may have a crucial role in tumorigenesis and are also likely to stimulate tumor growth, as knocking down the neural generating capabilities of human CSCs markedly reduced the growth of xenograft tumors in a murine model [141].

Novel data revealed that the next source of neurons residing and infiltrating cancer tissue might be the central nervous system [142]. In PC, the neural progenitors expressing doublecortin from the brain subventricular zone are able to access the bloodstream after disrupting the blood–brain barrier, eventually reaching the tumor stroma, where they differentiate into new adrenergic neurons [142]. It is also worth mentioning that enteric neural progenitors possess a higher efficiency in generating neurons than brain-derived progenitors [143].

Noteworthy, human mesenchymal stem cells, which are of bone marrow origin, may be an alternative source of neurons in tumors [144]. Human mesenchymal stem cells, if residing under proper conditions, are able to differentiate into functional neurons [145]. The concept of human mesenchymal stem cells as a source of neurons in cancers is, in fact, understudied.

Lately, the occurrence of a tumor-associated neural switch has been observed, where sensory nerve fibers were found to differentiate into neo-adrenergic nerves in head and neck tumors [31]. De novo transdifferentiated adrenergic fibers may thus increase the overall sympathetic nerve number without the neurogenesis phenomenon.

Unfortunately, in the available literature, there are little data on the possible source of new neurons in PDAC up to this date. A recent study, however, reported a lack of neurogenesis in a PDAC murine model [86]. Moreover, no evidence of the presence of neural progenitors from the brain subventricular zone in PDAC tissue was found [86]. The relative contribution of axonogenesis was put into question by the same authors; nevertheless, the concepts of active localized sprouting of axon terminals and passive engulfment of pre-existing sympathetic nerves by the tumor were proposed instead [86].

In prostatic cancer, the coexistence of axonogenesis and neurogenesis has been confirmed to increase nerve density in the tumor stroma [146,147]. High nerve density was associated with worse recurrence-free and cancer-specific survival in prostate and colorectal cancer [147,148].

The molecular basis of both axonogenesis and neurogenesis is unclear; however, the coexistence of neurotrophic growth factors and cytokines (granulocyte colony-stimulating factor in prostate cancer [149]), along with axon guidance molecules, influence the neurogenic response of cancers [134]. In PDAC, human Schwann cells exhibit activation via proinflammatory IL-6 signaling [150]. Furthermore, PDAC stromal cells producing leukemia inhibitory factor—an IL-6 class cytokine—contribute to tumor-associated neural remodeling [151].

## 7. Nerve Number and Neural Density in PDAC

Data regarding nerve density in PDAC are unclear, as nerve density in some reports was found to be decreased [152,153], whereas other authors suggested the opposite [118,154]. Furthermore, Iwasaki et al. demonstrated that the distribution of the nerves within the tumor is not even. Both nerve density and nerve amount in PDAC tend to decrease toward the center of the tumor, where the nerves are fully replaced, due to the desmoplastic change characteristic of PDAC [152]. A similar tendency has been observed in prostate cancer [155]. It suggests that regarding PDAC, nerve hypertrophy may be the predominating phenomenon, not de novo innervation. Therefore, it is more likely that nerve density, when measured exactly within PDAC tissue and not in the tumor surrounding area, is decreased in PDAC [152].

However, at the invasive tumor front (ITF) in PDAC, the tumor budding phenomenon, defined as the presence of isolated single cells or small clusters of up to five cells in the stroma, has been observed [156]. Increased distribution of parasympathetic fibers was found to be correlated with a high tumor budding number, early recurrence, and diminished survival [156]. Nevertheless, how the parasympathetic nerve fiber amount increases at the ITF is yet to be determined.

Parallelly, muscarinic acetylcholine receptor 3 (M3R), whose overexpression has been found to correlate with cancer progression and tumor metastasis in several malignancies [157,158,159], exerts an influence on poor prognosis in patients with PDAC [160]. Patients with high M3R expression more frequently possessed higher stage, lymph node metastasis, and shorter overall survival in comparison to the low expression [160].

Increased expression of M3R was observed at the ITF, among tumor budding cells, and in metastatic lymph nodes of PDAC specimens, whereas M3Rs were absent in adjacent noncancerous pancreatic tissue [160]. Moreover, upregulation of M3Rs concerned PDAC cells encircling or invading parasympathetic nerve fibers, thus suggesting the occurrence of nerve–cancer cell crosstalk, through which ACh released from cholinergic nerve fibers could contribute to the PDAC progression [160].

As shown in the stomach, stimulating the ACh–NGF axis may be sufficient to cause gastric cancer, where parasympathetic stimulation induces NGF expression within the stomach, promoting cholinergic axonogenesis and tumorigenesis [161]. Thus, it may be useful to decipher the exact role of the Ach–NGF axis in PDAC, as NGF signaling is known to increase cancer cell growth, PNI, and nerve density in this malignancy [154,162].

Additionally, extrapancreatic neuropathy, defined as changes in nerve trunk number, the proportion of neuritis, and difference in the distribution of sympathetic and parasympathetic nerves in extrapancreatic nerve plexus, was measured by Lu et al., who divided PDAC patients into two clusters: early and non-early metastasis, depending on the onset of liver metastasis [163]. Patients with early liver metastasis had a significantly higher mean nerve trunk number than those in the non-early liver metastasis group. Moreover, the early liver metastasis group held a higher proportion of neuritis, characterized by any neural inflammatory infiltration, and also had a higher concentration of both sympathetic and parasympathetic nerve fibers than non-early liver metastasis PDAC patients [163]. These findings may suggest that neurogenesis presumably would be apart from the cancer center, at the ITF of PDAC, or where PCCs reach extrapancreatic plexus niches.

Although it has not been suggested by other authors yet, increased sympathetic and parasympathetic innervation at distant sites in PDAC could be due to the nerve phenotype reprogramming. In oral cavity squamous cell cancers, the presence of TP53 mutations can translate into sensory-to-adrenergic transdifferentiation via cancer-derived miRNA-laden exosomes [31]. As mutations in the TP53 gene can be found in up to 70% of PDAC [164], it is not inconceivable to assume that TP53 loss may likewise lead to the adrenergic switch likewise. Therefore, conducting research on that topic regarding PDAC would be valuable and justified.

## 8. Conclusions and Future Directions

The prognosis of PDAC is uniformly poor despite substantial advances in the understanding of its biology and genetics. As PDAC is diagnosed with the advanced stage in most cases, only 20% of patients undergo surgical resection [165]. However, the estimated 5-year overall survival for this group is only 18% [166].

Recent insights into crosstalk between PCCs and the tumor microenvironment have shed new light on neural involvement in the initiation and progression of PDAC. Although the interdependence between the neural component and cancer cells in the pancreatic TME is complex and has not been completely elucidated, it presents itself as a promising future target area for anti-PDAC therapy.

In the case of omnipresent PNI in PDAC, a new quantification with a distinction between PNI and ENI could help stratify the risk of relapse and mortality for patients with this cancer. As PNI is induced by neurotrophic properties of the pancreatic microenvironment, a better understanding of nerve–cancer cell interactions may bring novel treatment modalities. Regrettably, evaluation of PNI takes place after surgical resection; thus, to use it as a potential predictive biomarker before surgery, developing novel surgical protocols for PDAC management, to diminish the high recurrence rates, should be introduced. The studies focusing on nerve density and nerve hypertrophy in PDAC have brought ambiguous conclusions, so clarification in this field is needed.

Recently, the dynamic evolution of knowledge regarding the role of neurogenesis and axonogenesis in numerous malignancies has been observed. It is not clear to what extent these phenomena affect PDAC development and progression and any attempt to unravel the complexity of the neural niche in pancreatic cancer may warrant further in-depth investigation.

It is imperative to foster an interdisciplinary approach, as cancer neurobiology lies at the crossroads between oncology and neuroscience for the achievement of substantial improvement in PDAC patients’ outcomes.
